# 原发免疫性血小板减少症合并血栓/栓塞诊断与防治中国专家共识（2023年版）

**DOI:** 10.3760/cma.j.issn.0253-2727.2023.01.002

**Published:** 2023-01

**Authors:** 中华医学会血液学分会血栓与止血学组

原发免疫性血小板减少症（primary immune thrombocytopenia, ITP）是常见的获得性出血性疾病。不同程度的出血是ITP的主要临床表现，轻至皮肤/黏膜出血，重至重要脏器的致命性出血。然而，部分患者也面临发生血栓/栓塞的风险[1]–[2]。心、脑等重要脏器的栓塞严重影响患者的生活质量并危及生命，血小板减少相关的出血高风险给抗凝治疗的顺利实施带来巨大挑战。目前尚缺乏针对ITP合并血栓/栓塞防治的指南或共识。中华医学会血液学分会血栓与止血学组组织国内专家制定本共识，旨在为预防ITP患者血栓事件的发生及规范血小板减少合并血栓/栓塞的诊治提供临床指导。

一、ITP合并血栓/栓塞的流行病学特征

西方学者观察到ITP患者动、静脉血栓事件年发生率分别为1.0～2.8/人、0.4～0.7/人，非ITP对照人群动、静脉血栓事件年发生率分别为0.7～1.8/人、0.1～0.4/人[3]–[7]。

我国规模最大的一项回顾性研究显示，ITP患者动脉血栓发生率（1.12％）高于静脉血栓发生率（0.22％），两者发生率均高于国内普通人群[2]。

性别差异对血栓形成的影响报道不一[8]–[9]。我国研究显示总血栓事件发生率无性别差异[1]–[2]。

二、ITP合并血栓/栓塞的发病机制

具体机制尚未明确。目前认为，ITP疾病本身、ITP治疗相关因素以及合并症等因素共同参与了血栓事件的发生。

（一）ITP疾病本身

血小板动力学研究显示慢性ITP患者血小板更新加速，外周血中具有更高比例的年轻血小板，其活性更强，更易发生黏附聚集。针对血小板膜糖蛋白（GPⅡb/Ⅲa、PⅠb/Ⅸ/Ⅴ）的自身抗体抗可通过结合FcγRⅡA进而活化血小板。同时，ITP患者体内血小板微颗粒及红细胞微颗粒明显增多，这些微颗粒表面表达丰富的磷脂酰丝氨酸，内部含有大量巨大血管性血友病因子（VWF）多聚体，对启动凝血、促进凝血酶生成及血栓形成发挥重要作用[10]–[15]。

血管内皮细胞损伤可以介导血小板激活及脂质斑块的形成，在冠心病、糖尿病、脑血管疾病中发挥了重要的致病作用[16]–[18]。研究证实ITP患者血浆中多种血管内皮细胞活化标志物水平升高，包括细胞间黏附分子1（ICAM-1）、血管细胞黏附分子（VCAM）、凝血酶调节蛋白（TM）、血管内皮生长因子（VEGF）、血管生成素2（Ang-2）等[19]–[20]，提示ITP患者具有血管内皮细胞损伤，具备血栓形成的病理基础。

另外，补体激活及抗凝因子水平降低也可能参与部分ITP患者高凝状态的形成[21]。

（二）ITP治疗相关因素

与血栓形成可能有关的ITP相关治疗主要包含糖皮质激素、静脉注射免疫球蛋白（IVIg）、重组人血小板生成素（rhTPO）、血小板生成素受体激动剂（TPO-RA）和脾切除。在短期内快速提升血小板水平是上述治疗方式的共同特点。此外，糖皮质激素可提升凝血因子Ⅷ水平、抑制纤溶，诱发高凝状态[22]。大剂量IVIg可增加血液黏稠度、影响血管内皮细胞、介导血管痉挛收缩[23]–[25]。

rhTPO及TPO-RA作为ITP二线治疗的主要药物，广泛用于糖皮质激素治疗无效或复发患者。研究显示，应用rhTPO或TPO-RA治疗的患者动/静脉血栓事件的发生率是未接受相应治疗ITP患者的2～3倍。血栓多发生于rhTPO或TPO-RA治疗的第1年，具有1个或以上血栓危险因素（高血压、吸烟、肥胖、血栓家族史等）的ITP患者是高发人群[26]–[27]。

脾切除后血栓风险增加的原因包括：血小板计数升高并活化增多、术后高凝状态、内皮细胞损伤及纤维蛋白原和纤溶酶原激活物抑制物-1（PAI-1）水平升高等。随着切脾时间延长，血栓风险逐渐上升，脾切除后10、20、30年的血栓事件发生率分别为11％、15％、21％[28]。因此，对脾切除术后血小板计数上升过高、过快者需进行血栓风险评估，对中高危患者给予血栓预防治疗[29]。

（三）合并症

合并糖尿病、高血压、心血管疾病、肿瘤、遗传性或获得性抗凝蛋白缺乏等疾病，可能增加ITP患者的血栓风险[30]–[31]。

三、ITP合并血栓/栓塞的危险因素

受人种、地域及纳入患者基线特征等因素的影响，不同研究对ITP合并血栓/栓塞的危险因素的分析结果有所差异。法国一项真实世界研究[32]发现，与动脉血栓相关的危险因素包括高龄、男性、心血管疾病史、脾切除史、既往应用rhTPO/TPO-RA及IVIg；与静脉血栓相关的危险因素包括高龄、血栓病史、肿瘤病史、脾切除史、既往应用糖皮质激素、rhTPO/TPO-RA及IVIg。我国的研究资料显示，年龄>40岁、合并≥2个心血管危险因素（糖尿病、高血压、血脂异常、吸烟）、血栓病史、≥3线ITP治疗、既往糖皮质激素治疗、脾切除与血栓发生存在显著相关性[1]–[2]。日本一项回顾性研究[30]证实，吸烟、高血压、男性、血栓病史、合并房颤与新诊断ITP患者发生血栓密切相关，其中吸烟及合并房颤是血栓发生的独立危险因素。

低中滴度的单一抗磷脂抗体单次阳性在ITP发生血栓/栓塞中的意义未明，需定期监测随访。

目前缺乏ITP患者发生血栓/栓塞的危险预测模型。在借鉴动脉粥样硬化血栓风险因素以及Caprini评分表[33]–[35]的基础上，结合ITP的疾病特点，总结已有ITP患者血栓发生风险因素评估的研究结果，本共识分别建立了ITP合并动、静脉血栓/栓塞风险评估量表（表1、表2），旨在为高危人群的筛选及后续ITP相关治疗方案的制定提供指导。根据量表中各项指标的合计评分，分为低危（≤9分）和高危（>9分）。此评估量表的临床应用价值尚需开展前瞻性、大样本、多中心的临床研究予以验证。

**表1:** 原发免疫性血小板减少症合并动脉血栓/栓塞风险评估量表

分值	项目
1分	男性，吸烟，高血压，糖尿病，肾功能不全（肾小球滤过率<60 ml/min），充血性心力衰竭，外周血管病变
2分	静脉注射免疫球蛋白（IVIg）、重组人血小板生成素（rhTPO）、血小板生成素受体激动剂（TPO-RA）治疗
3分	年龄≥75岁，脾切除史，动/静脉血栓病史（≥3个月），冠状动脉搭桥术后
5分	动/静脉血栓（<3个月）

**表2:** 原发免疫性血小板减少症合并静脉血栓/栓塞风险评估量表

分值	项目
1分	病史：年龄41～60岁，肥胖（身体质量指数≥25 kg/m^2^），炎症性肠病史，下肢水肿/静脉曲张，卧床，妊娠期或产后（1个月），脓毒症（<1个月），肺炎及严重肺病（<1个月）；外伤、手术、药物治疗：小手术，口服避孕药或激素替代治疗，糖皮质激素治疗
2分	病史：年龄61～74岁，恶性肿瘤（<5年），卧床（>72 h），石膏固定；外伤、手术、药物治疗：大手术（>45 min），腹腔镜手术（>45 min），关节镜，静脉注射免疫球蛋白（IVIg），重组人血小板生成素（rhTPO），血小板生成素受体激动剂（TPO-RA）
3分	病史：年龄≥75岁，脾切除史，动/静脉血栓病史（≥3个月），血栓性疾病家族史；实验室检查：血清同型半胱氨酸升高，蛋白C活性降低，蛋白S活性降低，抗凝血酶活性降低
5分	病史：动静脉血栓（<3个月）；外伤、手术、药物治疗：选择性关节置换术，急性脊髓损伤（<1个月），髋关节、骨盆或下肢骨折

四、ITP合并血栓/栓塞的临床特点

据统计，多数ITP患者发生血栓事件的时间在ITP诊断后15～25个月，少数患者以血栓为首发症状。发生血栓事件时中位血小板计数为（33～102）×10^9^/L。62％～70％的血栓事件发生于血小板计数<50×10^9^/L、28％～46％发生于血小板计数<30×10^9^/L时。部分患者可表现为出血和血栓并存。ITP患者动脉血栓/栓塞多发生于颅脑和心脏，表现为缺血性脑卒中和急性心肌梗死，少见外周动脉栓塞。ITP合并急性心肌梗死患者中，男性多于女性，65～79岁高发。高血压、高脂血症及糖尿病是ITP发生急性心肌梗死患者最常见的合并症[36]–[38]。静脉血栓/栓塞以下肢深静脉血栓多发，偶见肺栓塞。肠系膜静脉、门静脉血栓主要发生于脾切除术后。少数患者动静脉血栓/栓塞同时发生。研究显示，发生心肌梗死、缺血性脑卒中、深静脉血栓或肺栓塞可增加ITP患者一年内的死亡风险[39]。

五、ITP合并血栓/栓塞的诊断

（一）明确ITP诊断，排除其他可引起血小板减少并血栓/栓塞事件的疾病

需要排除结缔组织病（抗磷脂综合征、系统性红斑狼疮、系统性血管炎等）、肿瘤、血栓性血小板减少性紫癜（TTP）、Evans综合征、弥散性血管内凝血（DIC）、阵发性睡眠性血红蛋白尿症（PNH）、肝素诱导性血小板减少症（HIT）等。妊娠期患者还需注意排除HELLP综合征、子痫等。

（二）结合临床表现、实验室及影像学检查明确相应部位的血栓/栓塞诊断

1. 缺血性脑卒中：参照《中国急性缺血性脑卒中诊治指南2018》[40]。

2. 急性心肌梗死：参照《急性ST段抬高型心肌梗死诊断和治疗指南（2019）》[41]。

3. 肺栓塞（PE）：参照《急性肺栓塞多学科团队救治中国专家共识》[42]。

4. 深静脉血栓形成（DVT）：常发生于下肢。主要表现为患侧下肢肿胀、疼痛，可伴有皮肤颜色改变。小腿后方或大腿内侧压痛，活动后加重，抬高患肢可缓解。下肢静脉加压超声显像是诊断DVT的首选方法。

5. 门静脉血栓形成（PVT）：指发生于门静脉主干、肠系膜上静脉、肠系膜下静脉或脾静脉的血栓。门静脉血栓形成临床表现变化较大。血栓形成缓慢，可无明显症状或仅有轻微的非特异性临床表现。血栓形成呈急性或亚急性发展时，表现为腹痛和消化道出血，严重时可并发肠系膜静脉血栓形成，50％的患者有恶心和呕吐，少数患者有腹泻或便血。如病情进一步发展可出现肠梗阻及肠坏死的表现。ITP患者出现腹痛、黑便时需要注意鉴别消化道出血由血小板减少引起还是门静脉或肠系膜静脉血栓。腹部血管超声和CT有助于诊断，计算机断层扫描静脉造影（CTV）和MRI/磁共振静脉造影（MRV）为主要的确诊方法。

六、ITP合并血栓/栓塞的防治

ITP血栓/栓塞的防治，预防重于治疗。目前关于血小板减少患者进行血栓/栓塞的防治的安全性数据有限。对于血栓/栓塞高风险或已发生血栓/栓塞的ITP患者，需评估血栓/栓塞预防或抗栓治疗的风险及获益，兼顾患者意愿制定个体化的ITP相关治疗及抗凝/抗血小板策略（图1）。

**图1:**
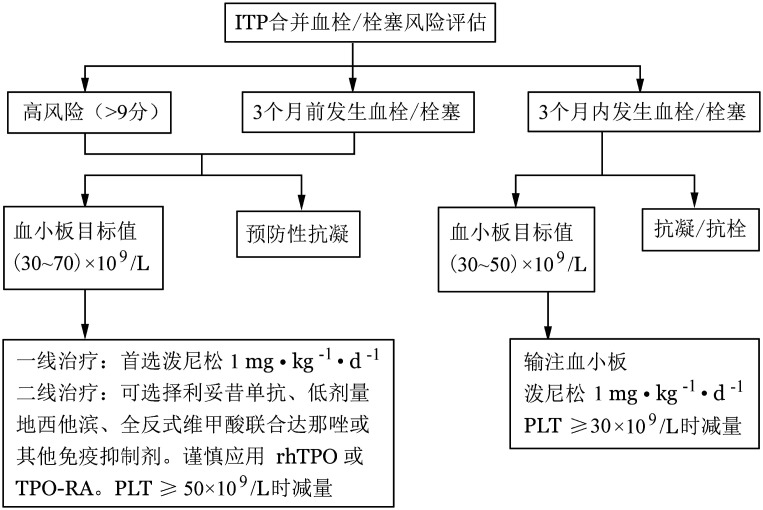
原发免疫性血小板减少症（ITP）合并血栓/栓塞防治流程

（一）血栓/栓塞高风险（>9分）或动静脉血栓/栓塞病史（≥3个月）

1. ITP相关治疗：鉴于ITP患者合并血栓/栓塞的风险高于普通人群，且以动脉血栓多发，推荐开始ITP相关治疗前，根据《ITP合并动、静脉血栓/栓塞风险评估量表》进行风险评估，对高危（>9分）或既往3个月前发生过动静脉血栓/栓塞的患者，建议下调血小板的治疗目标值并调整ITP治疗方案。

（1）血小板计数目标值：（30～70）×10^9^/L[43]，达标后口服阿司匹林（75～100）mg/d[44]。

（2）升血小板治疗：①一线治疗：建议首选泼尼松1 mg·kg^−1^·d^−1^，血小板计数≥（30～70）×10^9^/L时减量，6～8周内停用。②二线治疗：可选择利妥昔单抗、低剂量地西他滨、全反式维甲酸联合达那唑或其他免疫抑制剂。谨慎应用rhTPO和TPO-RA，从低剂量开始治疗，密切监测血常规，调整药物剂量，血小板计数≥50×10^9^/L时减量，维持血小板计数（30～70）×10^9^/L。

2. 预防性抗凝治疗：对合并心房颤动的患者，需由心内科专科医师评估是否具备预防性抗凝指征并参与制定抗凝方案。

（二）近期发生动静脉血栓/栓塞（< 3个月）

1. ITP相关治疗：ITP患者发生急性血栓/栓塞时，需要动态评估患者血栓进展或复发及出血的风险。如血小板计数未达到抗凝/抗血小板治疗要求的安全水平，可输注血小板[45]。血小板不易获得或输注无效时，可口服泼尼松1 mg·kg^−1^·d^−1^治疗，不建议应用大剂量地塞米松、IVIg、rhTPO及TPO-RA。治疗期间密切监测血小板计数，≥30×10^9^/L时减量，≥50×10^9^/L时停药。如出现明显的出血症状，应调整抗凝方案、积极控制出血。

如在ITP治疗过程中发生血栓/栓塞，建议组织多学科会诊，对患者目前血小板计数下的出血风险和血栓进展风险进行评估、权衡，决定是否快速减停当前ITP相关治疗或调换其他方案并决定抗凝/抗血小板治疗方案。

2. 抗凝/抗血小板治疗：由血液科与心内科、呼吸科、血管外科、神经内科等相关科室的专科医师共同协商制定治疗方案。

（三）启动抗栓药物治疗的血小板水平[43]

单一抗血小板或抗凝治疗：血小板计数≥（30～50）×10^9^/L；抗血小板联合抗凝治疗：血小板计数≥（50～70）×10^9^/L。

七、ITP合并血栓/栓塞的预后

血栓/栓塞不仅影响ITP相关治疗的顺利开展，也严重影响患者的生活质量甚至危及生命。早期识别血栓/栓塞高风险人群，及时调整ITP的治疗目标及治疗方案，对血栓/栓塞防治，保障医疗安全具有重大意义。
